# Supplementary material for: A dual-targeted drug inhibits cardiac ryanodine receptor Ca2+ leak but activates SERCA2a Ca2+ uptake
Source: Life Sci Alliance. 2023 Nov 27;7(2):e202302278. doi: 10.26508/lsa.202302278 (PMC10681910; doi:10.26508/lsa.202302278)
Supplement: Supplementary file 1 [file LSA-2023-02278_Supplemental_Data_1.docx]

**Supplemental Information**

**Synthesis**

**General materials and methods.** All reagents and solvents were purchased from commercial sources and used without further purification. Flash chromatography was performed using Biotage Isolera ONE flash purification system with a cartridge and solvent gradient indicated. NMR spectra were recorded at ambient temperature on Agilent 400-MR spectrometer (MPI NAT Göttingen) at 400.06 MHz (^1^H) and 100.60 MHz (^13^C), and the chemical shifts are reported in ppm. All ^1^H spectra are referenced to tetramethylsilane (*δ* =0 ppm) using the signals of the residual protons of CHCl_3_ (7.26 ppm) in CDCl_3_, CHD_2_CN (1.94 ppm) in CD_3_CN, CHD_2_OD (3.31 ppm) in CD_3_OD or DMSO-*d_5_* (2.50 ppm) in DMSO-*d_6_*. ^13^C spectra are referenced to tetramethylsilane (*δ* = 0 ppm) using the signals of the solvent: CDCl_3_ (77.16 ppm), CD_3_CN (1.32 ppm), CD_3_OD (49.00 ppm) or DMSO-*d_6_* (39.52 ppm). Multiplicities of signals are described as follows: s = singlet, br. s = broad singlet, d = doublet, q = quartet, dq = doublet of quartets, m = multiplet or overlap of signals. Low resolution mass spectra (50 – 3500 m/z) with electro-spray ionization (ESI) were recorded on a Varian 500-MS spectrometer (Agilent) at MPI NAT Göttingen. High resolution mass spectra (ESI-HRMS) were recorded on a MICROTOF spectrometer (Bruker) equipped with ESI ion source (Apollo) and direct injector with LC autosampler Agilent RR 1200 in the Institute of Organic and Biomolecular Chemistry (Georg-August-Universität Göttingen).

Liquid chromatography: analytical HPLC was performed with Knauer AZURA liquid chromatography system. Analytical column: Knauer Eurosphere II 100-5, C18-H, 5 μm, 150×4.6 mm (unless otherwise stated); solvent A: H_2_O + 0.1% v/v TFA, solvent B: MeCN + 0.1% v/v TFA; temperature 25 °C. Analytical TLC was performed on MERCK ready-to-use plates with silica gel 60 (F_254_).

**General procedure 1 for the synthesis of *N*-hydroxypropanediamides**

Scheme 1. Synthesis of compounds with hydroxylamine residues.

**Compound** **2**

**Ethyl 3-(7-methoxy-2,3-dihydrobenzo[1,4-*f*]thiazepin-4(5*H*)-yl)-3-oxopropanoate**

To 50 mg of 7-methoxy-2,3,4,5-tetrahydrobenzo[1,4-*f*]thiazepine **1** (BLD Pharm, 0.26 mmol) in DCM (5 ml), 50 µl pyridine was added; then the reaction mixture was cooled to 0°C. After that, 1.5 eq. ethyl (chloroformyl)​acetate (BLD Pharm, 69 mg, 0.39 mmol) was added dropwise. The reaction mixture was stirred for 2 h at 0 °C. After warming up to room temperature, the reaction mixture was washed with sat. aq. NaHCO_3_, 1 M HCl and water. The organic layer was separated and dried over Na_2_SO_4_. The solvent was evaporated under reduced pressure on a rotary evaporator. The title compound was isolated by means of flash chromatography (Biotage® HP-Sfär Silica HC Duo 20 µm 10 g, gradient 5% to 50% MeOH in DCM) to afford the **2** as an yellowish solid (27 mg, 33% yield). (Marks et al., US7879840B2, 2011).

^1^H NMR (400 MHz, CDCl_3_): mixture of rotamers *δ* 7.50 (d, *J* = 8.5 Hz, 0.4H), 7.41 (d, *J* = 8.4 Hz, 0.6H), 7.12 (d, *J* = 2.9 Hz, 0.6H), 6.83 (d, *J* = 2.7 Hz, 0.4H), 6.74 (dd, *J* = 8.5, 2.8 Hz, 0.4H), 6.70 (dd, *J* = 8.5, 2.8 Hz, 0.6H), 4.73 (br.s, 1H), 4.63 (s, 1H), 4.14 (qd, *J* = 7.1, 1.7 Hz, 2H), 4.09 (br.s, 1H), 3.94 (br. s, 1H), 3.80 (s, 1.2H), 3.78 (s, 1.8H), 3.52 (s, 0.8H), 3.38 (s, 1.2H), 2.81–2.73 (m, 2H), 1.29 – 1.15 (m, 3H).

^13^C NMR (101 MHz, CDCl_3_): mixture of rotamers *δ* 219.1, 167.5, 167.4, 166.2, 165.1, 159.6, 159.4, 143.2, 142.6, 135.0, 134.0, 126.9, 126.2, 117.1, 116.5, 113.6, 112.5, 62.6, 62.0, 61.7, 61.5, 55.6, 55.5, 55.0, 53.7, 52.5, 51.0, 41.7, 41.7, 41.0, 35.0, 33.6, 14.1, 14.1, 14.0.

**SKTT34**

**3-(7-Methoxy-2,3-dihydrobenzo[1,4-*f*]thiazepin-4(5*H*)-yl)-3-oxopropanoic acid^1^**

To the solution of **2** (27 mg, 0.09 mmol) in EtOH (0.4 ml), 10 mg (0.25 mmol) NaOH in 0.4 ml H_2_O was added. The reaction mixture was stirred at rt for 1 h. After pH was adjusted to 2 with 6 N HCl, the product was extracted with DCM. The organic layer was dried over Na_2_SO_4_ and the solvent evaporated giving 15 mg (60%) of the title compound.

^1^H NMR (400 MHz, CDCl_3_): mixture of rotamers *δ* 7.53 (d, *J* = 8.4 Hz, 0.5H), 7.47 (d, *J* = 8.5 Hz, 0.5H), 7.10 (d, *J* = 2.8 Hz, 1H), 6.90 (d, *J* = 2.7 Hz, 1H), 6.80–6.73 (m, 1H), 4.77 (s, 1H), 4.15 (s, 1H), 3.98 (d, *J* = 16.8 Hz, 1H), 3.82 (s, 1.5H), 3.81 (s, 1.5H),3.52 (s, 1H), 3.32 (s, 1H), 2.86 – 2.73 (m, 2H).

^13^C NMR (101 MHz, CDCl_3_): mixture of rotamers *δ* 170.3, 169.1, 168.3, 168.2, 159.8, 159.7, 141.9, 141.0, 135.4, 134.5, 126.5, 126.0, 117.6, 116.8, 113.7, 113.3, 55.7, 55.6, 54.3, 52.9, 52.7, 51.3, 35.5, 35.0, 34.6, 33.3.

**Compound 3**

**3-(7-Methoxy-2,3-dihydrobenzo[1,4-*f*]thiazepin-4(5*H*)-yl)-3-oxo-*N*-(trityloxy)propanamide**

To the solution of **SKTT34** (50 mg, 0.18 mmol) in DMF (0.5 ml), *O*-tritylhydroxylamine (BLD Pharm, 85 mg, 0.31 mmol) and 77 mg (0.6 mmol) *N*,*N*-diisopropylethylamine (DIEA) were added followed by 102 mg *O*-(7-azabenzotriazol-1-yl)-*N,N,N',N'*-tetramethyluronium hexafluorophosphate (HATU, 0.27 mmol). The reaction mixture was stirred at room temperature for 1 h. After the solvent was evaporated, the title compound was isolated by means of flash chromatography (eluent Hex/EtOAc) as an yellowish solid. Yield – 58 mg (58%). The compound was used in the next step without further purification.

^1^H NMR (400 MHz, DMSO-*d*_6_) *δ* 7.41 – 7.12 (m, 16H), 6.96 (dd, *J* = 9.2, 2.9 Hz, 1H), 6.81 – 6.74 (m, 1H), 4.42 (d, *J* = 22.7 Hz, 2H), 3.77 – 3.67 (m, 3H), 3.59 (d, *J* = 17.8 Hz, 2H), 3.02 (d, *J* = 29.5 Hz, 2H), 2.70 – 2.59 (m, 2H).

ESI-MS, positive mode: *m/z* (rel. int., %) = 561 (100) [*M*+Na]^+^.

**GM1760**

***N*-Hydroxy-3-(7-methoxy-2,3-dihydrobenzo[1,4-*f*]thiazepin-4(5*H*)-yl)-3-oxopropanamide**

Compound **3** (58 mg, 0.11 mmol) was dissolved in 2 ml 2% TFA in DCM giving the yellow solution. Then 100 µl (77 mg, 0.48 mmol) triisopropylsilane was added, and the reaction mixture was stirred at rt for 5 min. After the solvent was evaporated, the title compound was isolated by means of flash chromatography (Biotage® HP-Sfär Silica HC Duo 20 µm 10 g, gradient 5% to 50% MeOH in DCM) to afford **GM1760** as an yellowish solid. Yield –13 mg, 36%.

^1^H NMR (400 MHz, DMSO-*d*_6_): 2:1 mixture of rotamers *δ* 10.49 (s, 1H), 7.42 (dd, *J* = 9.9, 8.5 Hz, 0.7H), 7.16 (d, *J* = 2.8 Hz, 0.3H), 6.98 (d, *J* = 2.9 Hz, 0.3H), 6.82 (dd, *J* = 8.5, 2.8 Hz, 0.7H), 6.77 (dd, *J* = 8.4, 2.9 Hz, 1H), 4.71 (s, 0.7H), 4.54 (s, 1.3H), 4.00 – 3.90 (m, 2H), 3.77 (s, 1H), 3.74 (s, 2H), 3.20 (s, 0.7H), 3.11 (s, 1.3H), 2.87 – 2.81 (m, 1.3H), 2.74 (t, *J* = 5.0 Hz, 0.7H).

^13^C NMR (101 MHz, DMSO-*d*_6_): 2:1 mixture of rotamers *δ* 167.0, 165.9, 163.3, 163.2, 158.9, 158.5, 143.6, 143.2, 142.7, 133.9, 133.4, 126.2, 126.1, 117.5, 116.4, 112.8, 112.4, 55.4, 55.2, 53.0, 51.8, 49.8, 34.6, 33.0.

ESI-MS, positive mode: *m/z* (rel. int., %) = 297 (100) [*M*+H]^+^, 319 [*M*+Na]^+^.

HRMS (m/z): [M+H]^+^ calcd. for M = C_13_H_16_N_2_O_4_S, 297.0904; found, 297.0904.

Scheme 2. Synthesis of 3-(4-methoxyphenoxy)propan-1-amine derivatives.

**Compound 4**

**3-((3-(4-Methoxyphenoxy)propyl)amino)-3-oxopropanoic acid**

was obtained from 3-(4-methoxyphenoxy)propan-1-amine (BLD Pharm, 226 mg, 1.25 mmol), ethyl (chloroformyl)​acetate (324 mg, 2.15 mmol) using two-step procedure as described for **SKTT34**. Overall yield – 227 mg (82 %).

^1^H NMR (400 MHz, CD_3_OD) *δ* 6.90 – 6.76 (m, 4H), 3.97 (t, *J* = 6.2 Hz, 2H), 3.73 (s, 3H), 3.40 (t, *J* = 6.8 Hz, 2H), 3.26 (s, 2H), 2.00 – 1.90 (m, 2H).

^13^C NMR (101 MHz, CD_3_OD) *δ* 171.4, 169.1, 155.4, 154.5, 116.5, 115.6, 67.1, 56.1, 42.7, 42.5, 37.8, 30.1.

ESI-MS, positive mode: *m/z* (rel. int., %) = 290 (100) [*M*+Na]^+^.

HRMS (*m/z*): [*M*+H]^+^ calcd. for M = C_13_H_17_NO_5_, 270.1336; found, 270.1327.

**SKTT352**

***N*^1^-Hydroxy-*N*^3^-(3-(4-methoxyphenoxy)propyl)malonamide**

was obtained from **4** (227 mg, 0.85 mmol) and *O*-tritylhydroxylamine (350 mg, 1.28 mmol) using two-step procedure as described for **GM1760** (Scheme 1). The product was isolated by preparative HPLC with gradient elution (B/A, 20/80 → 100/0). Overall yield – 67 mg (28%).

^1^H NMR (400 MHz, DMSO-*d*_6_) *δ* 10.49 (d, *J* = 1.6 Hz, 1H, OH), 8.89 (d, *J* = 1.6 Hz, 1H, NH), 8.12 – 8.00 (m, 1H, NH), 6.92 – 6.77 (m, 4H), 3.92 (t, *J* = 6.3 Hz, 2H), 3.69 (s, 3H), 2.88 (s, 2H), 1.82 (p, *J* = 6.6 Hz, 2H).

^13^C NMR (101 MHz, DMSO-*d*_6_) *δ* 166.3, 163.8, 153.3, 152.5, 115.4, 114.6, 65.6, 55.3, 40.9, 40.1, 39.9, 39.7, 39.5, 39.3, 39.1, 38.9, 35.8, 28.8, 17.5, 7.3.

ESI-MS, negative mode: m/z (rel. int., %) = 281 (100) [M-H]^-^.

HRMS (m/z): [M+H]^+^ calcd. for M = C_13_H_18_N_2_O_5_, 283.1288; found, 283.1291.

**Compound 5**

**Ethyl (*E*)-4-((3-(4-methoxyphenoxy)propyl)amino)but-2-enoate**

To 383 mg of 3-(4-methoxyphenoxy)propan-1-amine (BLD Pharm, 2.11 mmol) in DCM (5 ml), 60 µl DIEA was added; then the reaction mixture was cooled to 0°C. After that, ethyl (*E*)-4-bromo-2-butenoate (191 mg, 1.06 mmol) was added dropwise. The reaction mixture was stirred at rt for 15 min. The reaction mixture was washed with sat. aq. NaHCO_3_ and water. The organic layer was separated and dried over Na_2_SO_4_. The solvent was evaporated under reduced pressure. The title compound was isolated by means of flash chromatography (Biotage® HP-Sfär Silica HC Duo 20 µm 10 g, gradient 2% to 20% MeOH in DCM) to afford **5** as an yellowish solid (150 mg, 48% yield).

^1^H NMR (400 MHz, CDCl_3_) *δ* 6.98 (dt, *J* = 15.7, 5.5 Hz, 1H, CH), 6.82 (d, *J* = 0.6 Hz, 4H), 5.98 (dt, *J* = 15.7, 1.8 Hz, 1H, CH), 4.18 (q, *J* = 7.1 Hz, 2H, NCH_2_), 3.99 (t, *J* = 6.1 Hz, 2H, CH_2_), 3.75 (s, 3H, OCH_3_), 3.42 (dd, *J* = 5.5, 1.9 Hz, 2H, CH_2_), 2.81 (t, *J* = 6.8 Hz, 2H, CH_2_), 1.98 – 1.89 (m, 2H, CH_2_), 1.28 (t, *J* = 7.1 Hz, 3H, CH_3_).

^13^C NMR (101 MHz, CDCl_3_) *δ* 166.5, 153.9, 153.1, 146.8, 121.7, 115.6, 115.5, 114.7, 66.9, 60.4, 55.8, 50.5, 46.6, 29.9, 14.4.

ESI-MS, positive mode: *m/z* (rel. int., %) = 294 (100) [*M*+H]^+^.

HRMS (*m/z*): [*M*+H]^+^ calcd. for M = C_16_H_23_NO_4_, 294.1700; found, 294.1699.

**GM1806**

**(*E*)-4-((3-(4-Methoxyphenoxy)propyl)amino)but-2-enoic acid**

was obtained by saponification of **5** (150 mg, 0.5 mmol) as described for **SKTT34**. After pH was adjusted to **2** with 6 N HCl, the title product precipitated and was filtered out. Yield – 96 mg (72%) of white solid.

^1^H NMR (400 MHz, DMSO-*d*_6_) *δ* 6.92 – 6.78 (m, 5H), 6.17 (dt, *J* = 15.8, 1.4 Hz, 1H, CH), 3.99 (t, *J* = 6.1 Hz, 1H, CH_2_), 3.78 (dd, *J* = 6.4, 1.6 Hz, 1H, CH_2_), 3.68 (s, 3H, CH_3_), 3.07 – 2.98 (m, 2H, CH_2_), 2.14 – 2.05 (m, 2H, CH_2_).

^13^C NMR (101 MHz, DMSO-*d*_6_) *δ* 166.0, 153.5, 152.3, 137.7, 127.1, 115.4, 115.2, 114.6, 65.2, 55.4, 46.5, 43.7, 25.5.

ESI-MS, positive mode: *m/z* (rel. int., %) = 266 (100) [*M*+H]^+^.

HRMS (*m/z*): [*M*+H]^+^ calcd. for M = C_14_H_19_NO_4_, 266.1387; found, 266.1388.

**GM1812**

**(*E*)-*N*-hydroxy-4-((3-(4-methoxyphenoxy)propyl)amino)but-2-enamide**

was obtained from compound **GM1806** (Scheme 2, 49 mg, 0.13 mmol) and *O*-tritylhydroxylamine (36 mg, 0.13 mmol) using the two-step-procedure similar to that for **SKTT352** (Scheme 2). The product was isolated by preparative HPLC with gradient elution (B/A, 30/70 → 100/0). Overall yield – 20 mg (55%) of beige solid.

^1^H NMR (400 MHz, CD_3_OD) *δ* 6.90 – 6.82 (m, 4H), 6.81 – 6.72 (m, 1H, CH), 6.21 (d, *J* = 15.3 Hz, 1H, CH), 4.05 (t, *J* = 5.7 Hz, 2H, CH_2_), 3.89 – 3.83 (m, 2H, CH_2_), 3.74 (s, 3H, OCH_3_), 3.28 – 3.20 (m, 2H, CH_2_), 2.21 – 2.09 (m, 2H, CH_2_).

^13^C NMR (101 MHz, CD_3_OD) *δ* 163.9, 162.8, 162.4, 155.7, 153.9, 132.8, 128.4, 116.5, 115.7, 66.6, 56.1, 46.5, 27.3, 18.7, 17.3.

ESI-MS, positive mode: *m/z* (rel. int., %) = 281 (100) [*M*+H]^+^.

HRMS (*m/z*): [*M*+H]^+^ calcd. for M = C_14_H_20_N_2_O_4_, 281.1496; found, 281.1495.

Scheme 3. Synthesis of 3-[(4-Methoxyphenyl)thio]-1-propanamine derivatives.

**3-[(4-Methoxyphenyl)thio]-1-propanamine**

was prepared from 4-methoxybenzenethiol (BLD Pharm, 1.4 g, 10 mmol), 3-bromopropan-1-amine hydrobromide (BLD Pharm, 2.37 g, 11 mmol) and potassium carbonate (2.8 g, 20.3 mmol) in 60 ml ethanol. After the reaction mixture was refluxed over-night, it was poured into cold water (30 ml) and extracted with CHCl_3_ (4 × 25 ml). The extracts were combined and washed with water (3 × 30 ml) and dried over anhydrous Na_2_SO_4_. The solvent was evaporated *in vacuo* affording the title compound as a yellowish oil. Yield – 1.18 g (60%).

^1^H NMR (400 MHz, CD_3_OD) *δ* 7.37 – 7.30 (m, 2H), 6.90 – 6.83 (m, 2H), 4.84 (s, 2H, NH), 3.77 (s, 3H, OCH_3_), 2.86 (t, J = 7.2 Hz, 2H, CH_2_), 2.73 (t, *J* = 7.1 Hz, 2H, CH_2_), 1.71 (p, *J* = 7.1 Hz, 2H, CH_2_).

^13^C NMR (101 MHz, CD_3_OD) *δ* 160.5, 134.3, 127.7, 115.6, 55.8, 41.3, 33.9, 33.1.

ESI-MS, positive mode: m/z (rel. int., %) = 198 (100) [M+H]^+^.

**GM1816**

**(*E*)-4-((3-((4-methoxyphenyl)thio)propyl)amino)but-2-enoic acid**

was obtained from 3-[(4-methoxyphenyl)thio]-1-propanamine (140 mg, 0.71 mmol) and (*E*)-4-bromo-2-butenoate (43 mg, 0.35 mmol) using the two-step-procedure described for **GM1806.** The product isolated by preparative HPLC with gradient elution (B/A, 30/70 → 100/0). Overall yield – 34 mg (35%) of white solid.

^1^H NMR (400 MHz, CD_3_OD) δ 7.44 – 7.34 (m, 2H, H-Ar), 6.95 – 6.86 (m, 2H, H-Ar), 6.83 (dt, *J* = 15.7, 6.6 Hz, 1H, CH_2_**CH**=), 6.18 (dt, *J* = 15.7, 1.5 Hz, 1H, CH=), 3.82 (dd, *J* = 6.6, 1.5 Hz, 2H, **CH_2_**CH=), 3.78 (s, 3H, OCH_3_), 3.19 – 3.10 (m, 2H, CH_2_), 2.92 (t, *J* = 7.0 Hz, 2H, CH_2_), 1.98 – 1.86 (m, 2H, CH_2_).

^13^C NMR (101 MHz, CD_3_OD) *δ* 168.0 (CO), 160.9 (C-Ar), 137.3(CH=), 134.9 (CH-Ar), 129.3 (C-Ar), 126.3 (CH=), 115.8 (CH-Ar), 55.8 (CH_3_), 48.6 (**CH_2_**CH=), 47.4 (CH_2_), 33.2 (CH_2_), 26.8 (CH_2_).

ESI-MS, positive mode: *m/z* (rel. int., %) = 282 (100) [*M*+H]^+^.

HRMS (*m/z*): [*M*+H]^+^ calcd. for M = C_17_H_25_N_3_O_3_S, 282.1158; found, 282.1164.

**GM1817**

**(2*E*,2'*E*)-4,4'-((3-((4-methoxyphenyl)thio)propyl)azanediyl)bis(but-2-enoic acid)**

was obtained from 3-[(4-methoxyphenyl)thio]-1-propanamine (125 mg, 0.63 mmol) and (*E*)-4-bromo-2-butenoate (122 mg, 0.63 mmol) using the two-step-procedure described for **GM1806.** The product was isolated by preparative HPLC with gradient elution (B/A, 10/70 → 100/0). Overall yield – 64 mg (28%) of white solid.

^1^H NMR (400 MHz, DMSO-*d*_6_) *δ* 7.41 – 7.25 (m, 2H), 6.96 – 6.86 (m, 2H), 6.86 – 6.70 (m, 2H, CH), 6.13 (dd, *J* = 17.8, 15.7 Hz, 2H, CH), 3.90 (d, *J* = 34.9 Hz, 2H, CH_2_), 3.73 (s, 3H, OCH_3_), 3.00 (d, *J* = 45.5 Hz, 2H), 2.89 (q, *J* = 7.4, 6.9 Hz, 2H), 2.49 (p, *J* = 1.9 Hz, 2H), 1.84 (d, *J* = 16.4 Hz, 2H).

^13^C NMR (101 MHz, DMSO-*d*_6_) *δ* 166.4, 159.0, 137.9, 133.0, 132.9, 127.6, 125.5, 115.3, 55.7, 55.6, 51.6, 49.0, 32.0, 25.7.

ESI-MS, positive mode: *m/z* (rel. int., %) = 366 (100) [*M*+H]^+^.

Scheme 4. Synthesis of 1,4-benzodiazepine derivatives with 4-aminobutyl linker.

**Compound 6**

**2-(4-(7-methoxy-2,3-dihydrobenzo[1,4-*f*]thiazepin-4(5*H*)-yl)butyl)isoindoline-1,3-dione**

To a solution of **1** (0.9 g, 4.6 mmol) in anhydrous 1,4-dioxane (32 ml), 2-(4-bromobutyl)isoindoline-1,3-dione (BLD Pharm, 1.3 g, 4.6 mmol) and K_2_CO_3_ (635 mg, 4.6 mmol) were added, and the reaction mixture was refluxed overnight in the argon atmosphere. After the reaction mixture was filtered from the precipitate, 20 ml DCM and 20 ml water were added to the filtrate. The organic phase was separated, the aqueous layer extracted with DCM (2×5 ml). The combined organic solutions were dried over Na_2_SO_4_. The filtrate was evaporated, and the product isolated by flash column chromatography using Biotage® SNAP Ultra 50 g cartridge with SiO_2_ (gradient: ethyl acetate in hexane 20% – 80%). Yield – 1.1 g (61%) of yellowish oil

^1^H NMR (400 MHz, CDCl_3_) *δ* 7.85 – 7.78 (m, 2H), 7.73 – 7.66 (m, 2H), 7.42 (d, *J* = 8.4 Hz, 1H), 6.78 (d, *J* = 2.8 Hz, 1H), 6.66 (dd, *J* = 8.4, 2.8 Hz, 1H), 4.09 (s, 2H, NCH_2_), 3.77 (s, 3H, OCH_3_), 3.68 (t, *J* = 7.1 Hz, 2H, CH_2_), 3.33 – 3.26 (m, 2H, CH_2_), 2.67 (dd, J = 6.6, 3.3 Hz, 2H, CH_2_), 2.45 – 2.35 (m, 2H, CH_2_), 1.73 – 1.63 (m, 2H, CH_2_), 1.57 – 1.48 (m, 2H, CH_2_).

^13^C NMR (101 MHz, CDCl_3_) *δ* 168.5, 159.2, 134.1, 134.0, 133.7, 132.2, 127.8, 123.4, 123.3, 116.9, 112.4, 59.6, 58.3, 55.4, 51.4, 37.9, 30.2, 26.4, 24.7.

ESI-MS, positive mode: m/z (rel. int., %) = 397 (100) [M+H]^+^.

HRMS (m/z): [M+H]^+^ calcd. for *M* = C_22_H_24_N_2_O_3_S, 397.1580; found, 397.1583.

**Compound 7**

**4-(7-methoxy-2,3-dihydrobenzo[1,4-*f*]thiazepin-4(5*H*)-yl)butan-1-amine**

To the compound **6** (330 mg, 0.84 mmol) in 2 ml ethanol, hydrazine hydrate (ABCR, 96 mg, 1.9 mmol) was added. The reaction mixture was stirred at 50 °C for 30 min. Then 5 ml of water was added, and the aqueous solution was extracted with DCM (2×5 ml). The combined organic solutions were dried over Na_2_SO_4_. The filtrate was evaporated, and the product isolated by preparative HPLC with gradient elution (B/A, 20/80 → 100/0). Yield – 210 mg (94%) of yellowish oil.

^1^H NMR (400 MHz, CDCl_3_) *δ* 7.42 (d, *J* = 8.4 Hz, 1H), 6.97 (d, *J* = 2.8 Hz, 1H), 6.72 (dd, *J* = 8.5, 2.8 Hz, 1H), 4.27 (s, 2H, NCH_2_), 3.79 (s, 3H, OCH_3_), 3.48 – 3.40 (m, 2H, CH_2_), 3.08 (t, *J* = 6.1 Hz, 2H, CH_2_), 2.78 (dd, J = 6.6, 3.2 Hz, 2H, CH_2_), 2.58 (t, J = 6.1 Hz, 2H, CH_2_), 1.94 (p, *J* = 6.2 Hz, 2H, CH_2_), 1.77 (q, J = 6.3 Hz, 2H, CH_2_).

^13^C NMR (101 MHz, CDCl_3_) *δ* 159.2, 144.8, 133.7, 127.9, 117.1, 112.1, 77.5, 77.4, 77.2, 76.8, 59.6, 58.4, 55.5, 51.9, 42.1, 31.2, 30.2, 28.1, 25.0, 24.8.

ESI-MS, positive mode: m/z (rel. int., %) = 267 (100) [M+H]^+^.

HRMS (m/z): [M+H]^+^ calcd. for M = C_14_H_22_N_2_OS, 267.1526; found, 267.1527.

**GM1774**

**3-((4-(7-methoxy-2,3-dihydrobenzo[1,4-*f*]thiazepin-4(5*H*)-yl)butyl)amino)-3-oxopropanoic acid**

was obtained from compound **7** (300 mg, 1.13 mmol), ethyl (chloroformyl)acetate (170 mg, 1.12 mmol) using the two-step-procedure similar to that for **SKTT34** (Scheme 1). The product isolated by preparative HPLC with gradient elution (B/A, 30/70 → 100/0). Overall yield – 50 mg (12.5%).

^1^H NMR (400 MHz, DMSO-*d_6_*) *δ* 8.22 – 8.12 (m, 1H, NH), 7.50 (d, *J* = 8.5 Hz, 1H), 7.31 (d, *J* = 2.8 Hz, 1H), 6.97 (dd, *J* = 8.5, 2.8 Hz, 1H), 4.54 (s, 2H, NCH_2_), 3.79 (s, 3H, OCH_3_), 3.78 – 3.53 (m, 2H, CH_2_), 3.25 – 2.93 (m, 2H, CH_2_), 1.76 (m, 2H, CH_2_), 1.43 (q, *J* = 7.2 Hz, 2H, CH_2_).

^13^C NMR (101 MHz, DMSO-*d_6_*) *δ* 169.9, 166.2, 159.7, 135.9, 134.2, 128.2, 119.6, 115.4, 56.0, 43.0, 42.1, 40.9, 38.3, 38.2, 26.5, 21.1.

ESI-MS, positive mode: m/z (rel. int., %) = 353 (100) [M+H]^+^.

HRMS (m/z): [M+H]^+^ calcd. for M = C_17_H_24_N_2_O_4_S, 353.1530; found, 353.1536.

**GM1778**

**2-((4-(7-methoxy-2,3-dihydrobenzo[1,4-*f*]thiazepin-4(5*H*)-yl)butyl)amino)-2-oxoacetic acid**

was obtained from compound **7** (300 mg, 1.13 mmol) and methyl 2-chloro-2-oxoacetate (136 mg, 1.12 mmol) (170 mg, 1.12 mmol) using the two-step-procedure similar to that for **SKTT34**. The product isolated by preparative HPLC with gradient elution (B/A, 20/80 → 100/0). Yield – 210 mg (94%) of yellowish oil. Yield – 210 mg (94%) of yellowish oil. Overall yield – 145 mg (38%).

^1^H NMR (400 MHz, CD_3_OD) *δ* 7.56 (d, *J* = 8.5 Hz, 1H), 7.21 (d, *J* = 2.8 Hz, 1H), 6.98 (dd, *J* = 8.5, 2.7 Hz, 1H), 4.68 (s, 2H), 3.84 (s, 3H), 3.80 – 3.62 (m, 3H), 3.38 – 3.31 (m, 2H), 3.09 (d, *J* = 73.5 Hz, 0H), 1.85 (s, 2H), 1.71 – 1.58 (m, 2H).

^13^C NMR (101 MHz, CD_3_OD) *δ* 162.6, 161.7, 160.5, 136.6, 135.5, 129.5, 119.8, 116.6, 60.1, 56.2, 53.8, 49.4, 39.6, 27.2, 22.5.

ESI-MS, positive mode: *m/z* (rel. int., %) = 339 (100) [*M*+H]^+^.

HRMS (*m/z*): [*M*+H]^+^ calcd. for M = C_16_H_22_N_2_O_4_S, 339.1373; found, 339.1378.

**GM1783**

***N*^1^-hydroxy-*N*^2^-(4-(7-methoxy-2,3-dihydrobenzo[1,4-*f*]thiazepin-4(5*H*)-yl)butyl)oxalamide**

was obtained from compound **GM1778** (70 mg, 0.2 mmol) and *O*-tritylhydroxylamine (68 mg, 0.25 mmol) using the similar two-step-procedure, as for **GM1760** (Scheme 1). The product isolated by preparative HPLC with gradient elution (B/A, 5/95 → 80/20). Overall yield – 12 mg (17%) of beige solid.

^1^H NMR (400 MHz, DMSO-*d*_6_) *δ* 11.48 (s, 1H), 8.80 (t, *J* = 6.1 Hz, 1H), 7.51 (d, *J* = 8.5 Hz, 1H), 7.29 (d, *J* = 2.8 Hz, 1H), 6.98 (dd, *J* = 8.5, 2.8 Hz, 1H), 4.56 (s, 2H), 3.79 (s, 16H), 3.14 (q, *J* = 6.7 Hz, 2H), 2.99 – 2.87 (m, 2H), 1.69 (s, 2H), 1.48 (q, *J* = 7.2 Hz, 2H).

^13^C NMR (101 MHz, DMSO-*d*_6_) *δ* 159.8, 159.2, 156.8, 133.8, 127.8, 119.0, 115.0, 55.6, 37.9, 25.8, 20.9.

ESI-MS, positive mode: *m/z* (rel. int., %) = 354 (100) [*M*+H]^+^.

HRMS (*m/z*): [*M*+H]^+^ calcd. for M = C_16_H_23_N_3_O_4_S, 354.1482; found, 354.1485.

**GM1840**

**3-(7-methoxy-2,3-dihydrobenzo[1,4-*f*]thiazepin-4(5*H*)-yl)-*N*-(2-methylquinolin-8-yl)-3-oxopropanamide**

To the solution of **SKTT34** (35 mg, 0.12 mmol) in DMF (0.5 ml), 2-methylquinolin-8-amine (24 mg, 0.15 mmol) and 46 mg (0.36 mmol) *N*,*N*-Diisopropylethylamine (DIEA) were added followed by 47 mg HATU (0.12 mmol). The reaction mixture was stirred at room temperature for 30 min. After the solvent was evaporated, the title compound was isolated by means of flash chromatography (Biotage® HP-Sfär Silica HC Duo 20 µm 10 g, gradient 12% to 100% EtOAc in hexane) to afford the title compound as a yellowish oil (30 mg, 59% yield).

^1^H NMR (400 MHz, DMSO-*d*_6_) *δ* 8.56 (td, *J* = 7.7, 1.3 Hz, 1H), 8.24 (dd, *J* = 8.4, 2.5 Hz, 1H), 7.58 (ddd, *J* = 8.3, 2.5, 1.3 Hz, 1H), 7.48 (ddd, *J* = 8.0, 4.0, 2.7 Hz, 2H), 7.39 (dd, *J* = 23.1, 8.4 Hz, 1H), 7.14 (dd, *J* = 62.0, 2.8 Hz, 1H), 6.74 (ddd, *J* = 15.9, 8.4, 2.9 Hz, 1H), 4.80 (s, 1H), 4.63 (s, 1H), 4.08 – 3.97 (m, 3H), 3.81 (d, *J* = 32.9 Hz, 2H), 3.74 (d, *J* = 1.6 Hz, 3H), 3.35 (s, 2H), 2.87 (dt, *J* = 21.1, 4.9 Hz, 2H), 2.71 (d, *J* = 7.5 Hz, 3H).

^13^C NMR (101 MHz, DMSO-*d*_6_) *δ* 167.7, 166.8, 166.7, 165.6, 165.5, 165.2, 165.1, 158.8, 158.6, 157.4, 157.4, 143.5, 142.9, 137.4, 137.4, 136.5, 133.9, 133.8, 133.8, 133.7, 133.5, 126.2, 126.0, 125.9, 125.9, 125.8, 122.8, 122.7, 121.7, 121.7, 117.6, 116.3, 116.2, 112.9, 112.4, 55.3, 55.2, 53.3, 52.8, 51.9, 49.9, 42.6, 42.3, 34.5, 32.9, 24.9, 24.9.

ESI-MS, positive mode: *m/z* (rel. int., %) = 422 (100) [*M*+H]^+^; 444 (70) [*M*+Na]^+^.

HRMS (*m/z*): [*M*+H]^+^ calcd. for C_23_H_23_N_3_O_3_S, 422.1533; found, 422.1517.

(1-(4-Bromobutyl)cyclopropoxy)(*tert*-butyl)dimethylsilane was obtained from in three steps from 5-(benzyloxy)pentan-1-ol (BLD Pharm, 2 g, 10.4 mmol) as described by Elek et al., *Org. Lett.* **2019,** *21* (20), 8473-8478.

**Compound 8**

**4-(1-((*tert*-butyldimethylsilyl)oxy)cyclopropyl)-*N*-(4-(1-((tert-butyldimethylsilyl)oxy)cyclopropyl)butyl)-*N*-(4-(7-methoxy-2,3-dihydrobenzo[1,4-*f*]thiazepin-4(5H)-yl)butyl)butan-1-amine**

To the suspension of NaH (60 % in mineral oil, 119 mg, 0.39 mmol) in 1.5 ml anhydrous DMF compound **7** (244 mg, 0.92 mmol) was added, and the reaction mixture was stirred at rt for 5 min. Then (1-(4-bromobutyl)cyclopropoxy)(*tert*-butyl)dimethylsilane was added and the reaction mixture was stirred overnight at rt. After the solvent was removed *in vacuo*, 5 ml water and 5 ml DCM were added. The organic solution was separated, the aqueous solution extracted with DCM (2×5 ml). The combined organic solutions were dried over Na_2_SO_4_. The filtrate was evaporated, and the product isolated by flash column chromatography using Biotage® SNAP Ultra 10g cartridge (gradient: methanol in DCM 4 % → 40 %). Yield – 63 mg (14 %) of yellowish oil.

^1^H NMR (400 MHz, CDCl_3_) *δ* 7.44 (d, *J* = 8.4 Hz, 1H, H_Ar_-5), 6.79 (d, *J* = 2.8 Hz, 1H, H_Ar_-8), 6.68 (dd, *J* = 8.4, 2.8 Hz, 1H, H_Ar_-6), 4.09 (s, 2H, NCH_2_), 3.79 (s, 3H, OCH_3_), 3.35 – 3.29 (m, 2H, CH_2_), 2.72 – 2.64 (m, 2H, CH_2_), 2.37 (t, *J* = 7.2 Hz, 8H, CH_2_), 1.51 – 1.33 (m, 16H, CH_2_), 0.84 (s, 18H, CH_3_), 0.71 – 0.62 (m, 4H, CH_2_), 0.42 – 0.35 (m, 4H, CH_2_), 0.08 (s, 12H, CH_3_).

^13^C NMR (101 MHz, CDCl_3_) *δ* 159.2, 145.2, 133.7, 127.9, 117.0, 112.0, 59.8 (CH_2_), 58.5 (CH_2_), 57.0 (CH_2_), 55.5 (OCH_3_), 39.2 (CH_2_), 30.4 (CH_2_), 25.9 (CH_3_), 24.1, 17.9, 13.2 (CH_2_), -3.3 (CH_3_).

ESI-MS, positive mode: *m/z* (rel. int., %) = 719 (100) [*M*+H]^+^.

HRMS (*m/z*): [M+H]^+^ calcd. for M = C_40_H_74_N_2_O_3_SSi_2_, 719.5031; found, 719.5033.

**GM1869**

**1,1*'*-(((4-(7-methoxy-2,3-dihydrobenzo[1,4-*f*]thiazepin-4(5*H*)-yl)butyl)azanediyl)bis(butane-4,1-diyl))bis(cyclopropan-1-ol)**

*t*-Butyldimethylsilyl deprotection of compound **8** (28 mg, 0.04 mmol) was performed in 200 µl 1 M tetrabutylammonium fluoride in THF. The reaction mixture was stirred overnight at rt. The title compound was isolated by preparative HPLC with gradient elution (B/A: 20/80 → 100/0). Yield 8 mg (41 %) of clear oil.

^1^H NMR (400 MHz, CD_3_OD) *δ* 7.55 (d, *J* = 8.5 Hz, 1H, H_Ar_-5), 7.21 (d, *J* = 2.8 Hz, 1H, H_Ar_-8), 6.98 (dd, *J* = 8.5, 2.8 Hz, 1H, H_Ar_-6), 4.68 (br. s, 2H, NCH_2_), 3.79 (s, 3H, OCH_3_), 3.76 – 3.68 (d, J = 19.5 Hz, 2H, NCH_2_), 3.24 – 3.06 (s, 10H, CH_2_), 1.97 – 1.74 (m, 8H, CH_2_), 1.67 – 1.55 (d, J = 3.6 Hz, 8H, CH_2_), 0.59 – 0.48 (m, 4H, CH_2_), 0.40 – 0.19 (m, 4H, CH_2_).

^13^C NMR (101 MHz, DMSO-*d_6_*) *δ* 159.2, 158.5, 158.2, 133.8, 127.8, 119.2, 114.9, 55.5, 53.5, 52.1, 51.1, 40.6, 37.4, 35.0, 23.0, 22.9, 20.7, 20.2, 12.8, 7.6.

ESI-MS, positive mode: m/z (rel. int., %) = 491 (100) [M+H]^+^.

HRMS (m/z): [M+H]^+^ calcd. for M = C_28_H_46_N_2_O_3_S, 491.3302; found, 491.3289.

**GM1891**

***N*^1^-(3-(4-methoxyphenoxy)propyl)-*N*^3^-(quinolin-8-yl)malonamide**

To 18 mg (67 µmol) of **4** in DMSO (1 ml), 50 µl Et_3_N was added, then ethyl 8-aminoquinoline (BLD Pharm, 12 mg, 84 µmol) and HATU (28 mg, 74 µmol) were added. The reaction mixture was stirred at rt for 20 min. Then the solvent was evaporated *in vacuo*, 20 ml DCM added, and the reaction mixture washed with water several times. The organic layer was separated and dried over Na_2_SO_4_. The solvent was evaporated under reduced pressure, and the title compound was isolated by flash chromatography (Biotage® HP-Sfär Silica HC Duo 20 µm 10 g, gradient 2% to 20% EtOAc in hexane) to afford the title compound as an yellowish solid (14 mg, 36% yield).

^1^H NMR (400 MHz, CDCl_3_) *δ* 10.61 (s, 1H, NH), 8.86 (dd, *J* = 4.2, 1.7 Hz, 1H, H-2′), 8.72 (dd, *J* = 6.0, 3.0 Hz, 1H, H-4′), 8.16 (dd, *J* = 8.3, 1.7 Hz, 1H, H-7′), 7.57 – 7.51 (m, 2H, H-5′, 6′), 7.46 (dd, *J* = 8.3, 8.3 Hz, 1H, H-3′), 7.36 (d, *J* = 5.1 Hz, 1H, NH), 6.89 – 6.74 (m, 4H, H_Ar_, H-2,3,5,6), 4.00 (t, *J* = 5.9 Hz, 2H, OCH_2_), 3.75 (d, *J* = 0.6 Hz, 3H, OCH_3_), 3.60 – 3.49 (m, 4H, CH_2_), 2.09 – 1.96 (m, 2H, CH_2_).

^13^C NMR (101 MHz, CDCl_3_) *δ* 166.6 (CO), 166.2 (CO), 154.0, 152.9, 148.7, 138.7, 136.4, 134.2, 128.1, 127.3, 122.4, 121.9, 117.0, 115.6, 114.8, 66.9 (OCH_3_), 55.8 (CH_2_), 45.0 (CH_2_), 37.7 (CH_2_), 29.1(CH_2_).

ESI-MS, positive mode: *m/z* (rel. int., %) = 416 (100) [*M*+Na]^+^.

HRMS (*m/z*): [*M*+H]^+^ calcd. for M = C_22_H_23_N_3_O_4_, 394.1761; found, 394.1758.

**GM1892**

***N*^1^-(3-(4-methoxyphenoxy)propyl)- *N*^3^-(2-methylquinolin-8-yl)malonamide**

was obtained similarly to **GM1891** (Scheme 2) from 3-((3-(4-methoxyphenoxy)propyl)amino)-3-oxopropanoic acid (**4**, 24 mg, 90 µmol) and 2-methylquinolin-8-amine (BLD Pharm, 18 mg, 114 µmol). The title compound was isolated by means of flash chromatography (Biotage® HP-Sfär Silica HC Duo 20 µm 10 g, gradient 22% to 100% EtOAc in hexane) to afford the title compound as an yellowish solid (24 mg, 66% yield).

^1^H NMR (400 MHz, CDCl_3_) *δ* 10.44 (s, 1H, NH), 8.67 (dd, *J* = 7.3, 1.7 Hz, 1H, H-7′), 8.04 (d, *J* = 8.4 Hz, 1H, H-4′), 7.61 – 7.41 (m, 3H, NH, H-5′,6′), 7.33 (d, *J* = 8.4 Hz, 1H, H-3′), 6.91 – 6.73 (m, 4H, H_Ar_, H-2,3,5,6), 4.00 (t, *J* = 5.9 Hz, 2H, OCH_2_), 3.75 (s, 3H, OCH_3_), 3.60 – 3.44 (m, 4H, CH_2_), 2.76 (s, 3H, CH_3_), 2.10 – 1.94 (m, 2H, CH_2_).

^13^C NMR (101 MHz, CDCl_3_) *δ* 166.4 (CO), 166.4 (CO), 157.8, 154.0, 153.0, 138.0, 136.5, 133.5, 126.2, 126.2, 122.8, 122.2, 116.9, 115.6, 114.7, 66.9 (OCH_3_), 55.9 (CH_2_), 45.1 (CH_2_), 37.6 (CH_2_), 29.2 (CH_2_), 25.4 (CH_3_).

ESI-MS, positive mode: *m/z* (rel. int., %) = 430 (100) [*M*+Na]^+^.

HRMS (*m/z*): [*M*+H]^+^ calcd. for M = C_23_H_25_N_3_O_4_, 408.1918; found, 408.1922.

**GM1893**

***N^1^*-(2-(3,5-dimethyl-1*H*-pyrazol-1-yl)quinolin-8-yl)-*N^3^*-(3-(4-methoxyphenoxy)propyl)malonamide**

was obtained similarly to **GM1891** (Scheme 2) from 3-((3-(4-methoxyphenoxy)propyl)amino)-3-oxopropanoic acid (**4**, 17 mg, 60 µmol) and 2-(3,5-dimethyl-1*H*-pyrazol-1-yl)quinolin-8-amine (Fluorochem, 19 mg, 80 µmol). The title compound was isolated by means of flash chromatography (Biotage® HP-Sfär Silica HC Duo 20 µm 10 g, gradient 22% to 100% EtOAc in hexane) to afford the title compound as an yellowish solid (24 mg, 66% yield).

^1^H NMR (400 MHz, CDCl_3_) *δ* 9.87 (s, 1H, NH), 8.69 (dd, *J* = 7.6, 1.4 Hz, 1H, H-7′), 8.25 – 8.15 (m, 2H, H-3′,4′), 7.55 – 7.41 (m, *J* = 7.9 Hz, 2H, NH, H-5′,6′), 6.88 – 6.73 (m, 4H, H_Ar_, H-2,3,5,6), 6.10 (d, *J* = 1.0 Hz, 1H, H-10′), 3.99 (t, *J* = 5.9 Hz, 2H, CH_2_), 3.75 (d, *J* = 7.6 Hz, 3H, OCH_3_), 3.59 – 3.49 (m, 2H, CH_2_), 3.46 (s, 2H, CH_2_), 2.87 (s, 3H, CH_3_), 2.34 (s, 3H, CH_3_), 2.02 (p, *J* = 6.3 Hz, 2H, CH_2_).

^13^C NMR (101 MHz, CDCl_3_) *δ* 166.1 (CO), 154.0 (CO), 153.0, 152.9, 151.2, 150.7, 141.7, 139.1, 136.5, 133.5, 126.3, 126.0, 122.4, 118.1, 115.8, 115.6, 114.8, 114.7, 110.4, 66.9 (OCH_3_), 55.8 (CH_3_), 45.2 (CH_3_), 37.7 (CH_2_), 37.6 (CH_2_), 29.1 (CH_2_), 15.6 (CH_2_).

ESI-MS, positive mode: *m/z* (rel. int., %) = 510 (100) [*M*+Na]^+^.

HRMS (*m/z*): [*M*+H]^+^ calcd. for M = C_27_H_29_N_3_O_4_, 488.2292; found, 488.2295.

**Compound 9**

***tert*-butyl (2-(3-(7-methoxy-2,3-dihydrobenzo[1,4-*f*]thiazepin-4(5*H*)-yl)-3-oxopropanamido)ethyl)carbamate**

To the solution of **SKTT34** (50 mg, 0.18 mmol) in DMSO (5 ml), *O*-(*N*-succinimidyl)-*N*,*N*,*N'*,*N'*-tetramethyluronium tetrafluoroborate (TSTU, 75 mg, 0.25 mmol) and 10 µl Et_3_N were added. After 10 min, *N*-(*tert*-butoxycarbonyl)-1,4-diaminobutane (51 mg, 0.27 mmol) was added, and the reaction mixture was stirred for 3 h at rt. Then the volatiles were evaporated *in vacuo*, and the title compound was isolated by means of flash chromatography (Biotage® HP-Sfär Silica HC Duo 20 µm 10 g, gradient 22% to 100% MeOH in DCM) as a yellowish oil (74 mg, 94% yield).

^1^H NMR (400 MHz, CDCl_3_) 1:1 mixture of rotamers: *δ* 7.47 (d, *J* = 8.5 Hz, 0.5H), 7.47 (d, *J* = 8.5 Hz, 0.5H), 7.08 (d, *J* = 2.8 Hz, 0.5H), 7.01 (d, *J* = 2.8 Hz, 0.5H), 6.77 – 6.69 (m, 1H), 4.75 (s, 1H, NCH_2_), 4.70 – 4.57 (m, 1H, NCH_2_), 4.05 (br. s, 2H, CH_2_), 3.82 (s, 1.5H, OCH_3_) 3.80 (s, 1.5H, OCH_3_), 3.33 – 3.02 (m, 6H, CH_2_), 2.86 – 2.76 (m, 2H, CH_2_), 1.75 – 1.57 (m, 2H, CH_2_ ), 1.49 – 1.32 (m, 12H, CH_2_, CH_3_).

ESI-MS, positive mode: *m/z* (rel. int., %) = 452 (100) [*M*+H]^+^.

**GM1938**

***N*-(4-aminobutyl)-3-(7-methoxy-2,3-dihydrobenzo[1,4-*f*]thiazepin-4(5*H*)-yl)-3-oxopropanamide**

74 mg (0.17 mmol) of **9** was dissolved in 10 % TFA/DCM mixture (1.5 ml), and the reaction mixture stirred at room temperature overnight. Then the solvent was evaporated *in vacuo,* and the title compound was isolated by preparative HPLC with gradient elution (B/A: 10/90 → 70/30). Yield 30 mg (50 %) of white solid.

1H NMR (400 MHz, CD_3_OD) 1:2 mixture of rotamers: *δ* 7.47 (d, *J* = 8.4 Hz, 0.33H), 7.42 (d, *J* = 8.4 Hz, 0.67H), 7.07 (d, *J* = 2.9 Hz, 0.67H), 7.04 (d, *J* = 2.9 Hz, 0.33H), 6.81 (dd, *J* = 8.4, 2.9 Hz, 0.33H), 6.74 (dd, *J* = 8.4, 2.9 Hz, 0.67H), 4.76 (s, 0.66H, NCH_2_), 4.69 (s, 1.34H, NCH_2_), 4.07 (br. s, 0.66H, CH_2_), 4.01 (br. s, 1.34H, CH_2_), 3.81 (s, 1H, OCH_3_), 3.78 (s, 2H, OCH_3_), 3.35 – 3.27 (m, 1.33H), 3.26 – 3.17 (m, 2.67H), 2.97 – 2.88 (m, 2H, CH_2_), 2.83 (q, *J* = 4.7 Hz, 1.34H, CH_2_), 2.78 (t, *J* = 5.0 Hz, 0.66H, CH_2_), 1.77 – 1.49 (m, 4H, CH_2_).

^13^C NMR (101 MHz, CD_3_OD) 1:2 mixture of rotamers: *δ* 213.2, 169.8, 169.5, 169.5, 168.8, 162.4, 161.0, 160.7, 144.5, 143.9, 135.5, 134.9, 128.1, 128.0, 118.5, 117.5, 113.9, 113.8, 56.0, 55.8, 55.2, 54.8, 53.5, 51.8, 41.8, 40.2, 39.5, 39.5, 36.5, 35.8, 34.3, 27.2, 25.6, 24.8, 22.9, 21.3, 13.9, 8.0.

ESI-MS, positive mode: *m/z* (rel. int., %) = 352 (100) [*M*+H]^+^.

HRMS (*m/z*): [*M*+H]^+^ calcd. for M = C_17_H_25_N_3_O_3_S, 352.1689; found, 352.1694.
